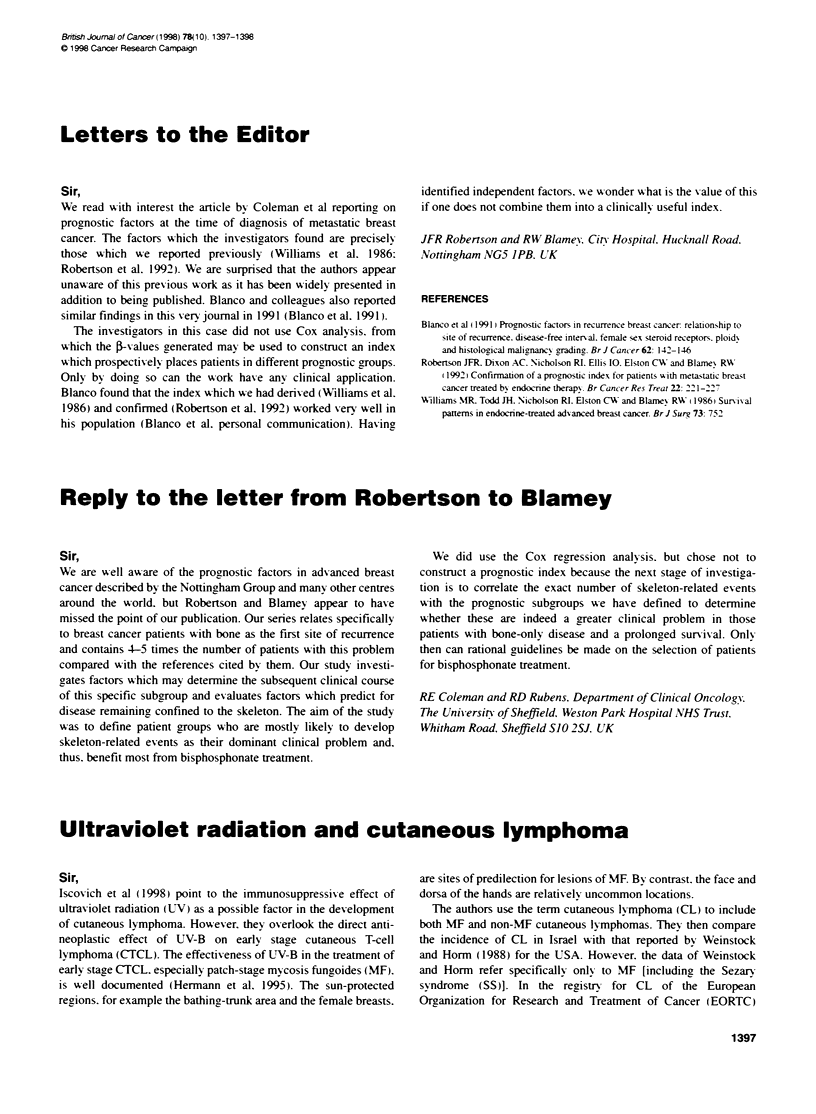# Reply to the letter from Robertson to Blamey

**Published:** 1998-11

**Authors:** 


### Reply to the letter from Robertson to Blamey

**Sir,**

We are well aware of the prognostic factors in advanced breast cancer described by the Nottingham Group and many other centres around the world, but Robertson and Blamey appear to have missed the point of our publication. Our series relates specifically to breast cancer patients with bone as the first site of recurrence and contains 4–5 times the number of patients with this problem compared with the references cited by them. Our study investigates factors which may determine the subsequent clinical course of this specific subgroup and evaluates factors which predict for disease remaining confined to the skeleton. The aim of the study was to define patient groups who are mostly likely to develop skeleton-related events as their dominant clinical problem and, thus, benefit most from bisphosphonate treatment.

We did use the Cox regression analysis, but chose not to construct a prognostic index because the next stage of investigation is to correlate the exact number of skeleton-related events with the prognostic subgroups we have defined to determine whether these are indeed a greater clinical problem in those patients with bone-only disease and a prolonged survival. Only then can rational guidelines be made on the selection of patients for bisphosphonate treatment.

*RE Coleman and RD Rubens, Department of Clinical Oncology, The University of Sheffield, Weston Park Hospital NHS Trust, Whitham Road, Sheffield S10 2SJ, UK*